# Current Epidemiological Assessment of Bancroftian Filariasis in Tanga Region, Northeastern Tanzania

**DOI:** 10.1155/2016/7408187

**Published:** 2016-12-06

**Authors:** Happyness J. Mshana, Vito Baraka, Gerald Misinzo, Williams H. Makunde

**Affiliations:** ^1^National Institute for Medical Research, Tanga Research Centre, P.O. Box 5004, Tanga, Tanzania; ^2^Sokoine University of Agriculture, Morogoro, Tanzania; ^3^Global Health Institute, University of Antwerp, Antwerp, Belgium

## Abstract

*Background*. Tanzania started a countrywide lymphatic filariasis elimination programme in 2000 adopting the mass drug administration (MDA) strategy. The drug used for the programme was the combination of ivermectin and albendazole. However, there is limited information on the current epidemiological trend of the infections, where MDA implementation is ongoing. The present study aimed at assessing the current status of Bancroftian filariasis infection rate and morbidity where MDA has been distributed and administered for over eight rounds.* Methodology*. The study was a cross-sectional descriptive study involving 272 individuals (>18 years) from endemic communities in Tanga region where MDA has been implemented. Clinical, sociodemographic, and circulating filarial antigen (CFA) test was undertaken using immune chromatographic card test according to the manufacturer's instructions.* Results*. A total of 472 individuals were screened: 307/472 (65.1%) were males while 165/472 (34.9%) were females. The overall prevalence of CFA was 5.51%, that of hydrocoele was 73.2%, and that of lymphoedema was 16.0%. The prevalence of hydrocoele combined with lymphoedema was 5.5%.* Conclusion*. Our findings demonstrate a considerable reduction in filarial infection. However, there is clear evidence of ongoing transmission despite the 8 rounds of MDA. It is unlikely that the annual MDA would interrupt filarial transmission; therefore, additional strategies are needed to accelerate lymphatic filariasis control and elimination.

## 1. Introduction

Lymphatic filariasis (LF) is the second leading cause of long-term disability globally due to lymphoedema, elephantiasis, and hydrocele [1]. The long-term disability is triggered through overt hydrocoele, lymphoedema, elephantiasis, repeated orchitis, and adenolymphangitis [2]. These acute and chronic pathologies impose a significant impediment to socioeconomic development and an extremely poor quality of life [3]. LF affects more than 120 million individuals globally [4]. It is estimated that around 20% of the world population in more than 83 countries are at risk of acquiring infection which is 1.1 billion people [5]. In the endemic communities, 7% of the adult population suffer from lymphoedema whereas 30–50% develop hydrocele [6]. In sub-Saharan Africa (SSA), the predominant parasite is filarial nematode* Wuchereria bancrofti (W. bancrofti)* which is estimated to affect 500 million people [7]. Epidemiological mapping in Tanzania has shown that the risk is particularly high in the coastal zone along the Indian Ocean although cases are also reported in other mainland regions [8].

In response to the World Health Organization (WHO) Global Programme to Eliminate Lymphatic Filariasis (GPELF) that aimed at filariasis elimination, Tanzania established the National Lymphatic Filariasis Elimination Programme (NLFEP) in 2000 where targeted communities were given a combination of ivermectin and albendazole (ALB+IV) annually [8]. The ivermectin dosage is administered according to body weight (150–200 *μ*g/kg) while 400 mg of albendazole is given to individuals aged ≥5 years in all endemic districts. In some settings, it has shown a reduction of infections rate to low levels as 1%. Conversely, the endemic areas along the coast of the Indian Ocean have shown a high prevalence of infection up to 63.3% despite the ongoing MDA for over eight years [8]. It was anticipated that using the MDA strategy as recommended in WHO guidelines on MDA coverage (>65%) through a variety of approaches will lead to interrupting transmission [3], whereby microfilariae (mf) levels will reach below 1%.

However, evidence in Tanga region has shown that, despite 8 rounds of MDA and supplementation with other interventions such as the provision of insecticide-treated nets (ITNs), still, both infection and clinical disease rates remain high especially along the coastline [8]. The prevalence in Tanga region before the MDA survey in 2004 of the community mf was 24.5%, that of CFA was 63.3%, and that of specific antibodies to recombinant filarial antigen was 78.9%. In comparison, after 8 rounds of MDA, the CFA and mf prevalence in combined study communities was reduced by 75.5% and 89.6%, respectively, compared to baseline levels, while the CFA prevalence in school children was reduced by 90.9% compared to baseline [8]. The present study was designed to ascertain the LF infections rates and morbidities after the MDA rounds in Tanga region.

## 2. Methods

### 2.1. Study Area and Population

The study was conducted in Tanga region in Northeastern Tanzania along the Indian Coast (05°04′S, 39°06′E). The region is characterised mainly by two rain seasons annually: the long rains from March to June and the less intensive short rains from November to December. The majority of the inhabitants practice subsistence farming, fishing, and livestock keeping. The climate in Tanga region is warm and wet. In most cases, there is no big variation of temperature at the coast due to the influence of the Indian Ocean. Also, the region is characterised by high humidity, which often goes up to 100% maximum and ranges from 65 to 70% minimum. There are health facilities in most villages in the regions, and the majority of the population have access to a health facility within a distance of 6 km. Most of the houses are made of mud walls with their roofs thatched with dried coconut leaves. This makes it easier for the vector to penetrate. Bancroftian filariasis control started in 2004 using MDA campaigns and advocacy with ivermectin and albendazole in all districts [8]. The estimated population size of the area was 2,045,205 inhabitants according to 2012 national census survey [9].

### 2.2. Study Design

The study was a cross-sectional, descriptive study conducted between April and May 2015. The study participants were selected using convenience sampling, a nonprobability sampling technique where individuals were selected based on their convenient accessibility and proximity.

### 2.3. Ethical Considerations

Ethical clearance for the study was granted by the Medical Research Coordinating Committee of the National Institute for Medical Research in Tanzania (NIMR-MRCC). The investigators explained the purpose of the study and all study participants consented orally and in writing.

### 2.4. Clinical Examination

A clinician experienced in LF examined those patients and recorded the swelling of the lower limbs and scrotum in stages according to Meyrowitsch et al. [10]. Thereafter, a semistructured questionnaire was used to collect the demographic information and other clinical disease conditions. Individuals with lymphoedema and hydrocoele stages lower than III were treated with antibiotic (doxycycline or tetracycline) for 7 days and topical broad-spectrum antifungal cream as recommended by the study physician.

### 2.5. Parasitological Examination

Patients were then asked to donate 100 *μ*L for circulating filarial antigen examination using immune chromatographic test cards. The BinaxNOW Filariasis immune chromatographic card test (Alere Inc., Scarborough, ME) was used to detect circulating filarial antigen as described in the WHO guidelines [11]. Briefly, 100 *μ*L of finger-prick blood was collected from each individual and then transferred to an immune chromatographic card test using a calibrated capillary tube. The test was read 10 minutes after closing the card, as per the manufacturer's instructions. The antigen contains the epitope present in circulating* W. bancrofti* antigen that was detected by the BinaxNOW Filariasis test. The identification number and test result of each individual tested were recorded in the case report form.

## 3. Results

**Figure 1:**
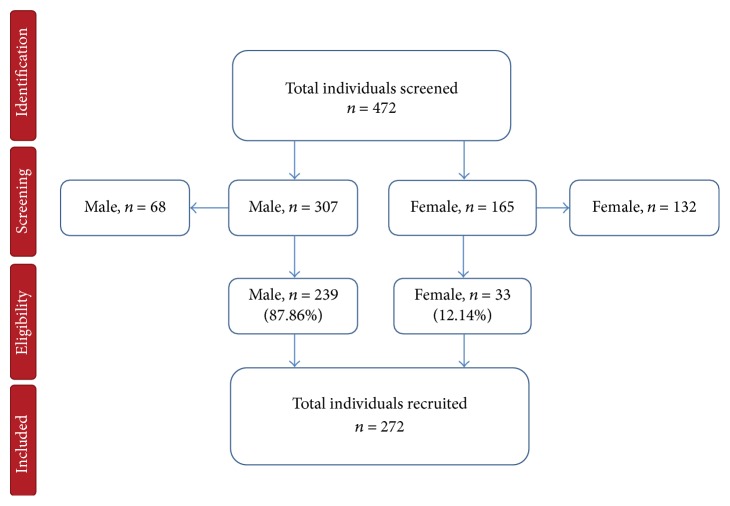
Study profile of the individuals recruited in the cross-sectional survey in Tanga region.

A total of 472 individuals were examined during the community survey, among whom 65.1% (307) were males while 34.9% (165) were females. Of those, 272 were recruited for the study, of whom 87.86% were males and 12.14% were females, *p* ≤ 0.0001. The rest did not fulfil the inclusion criteria as presented in the flow diagram (Figure 1). The study population that was more frequently observed with high proportion was the 32–45-year age group with a rate of 24.2%, followed by the 60–73-year age group with a rate of 33.1%, and the majority of them were fishermen (62.5%) (Table 1).

**Figure 2:**
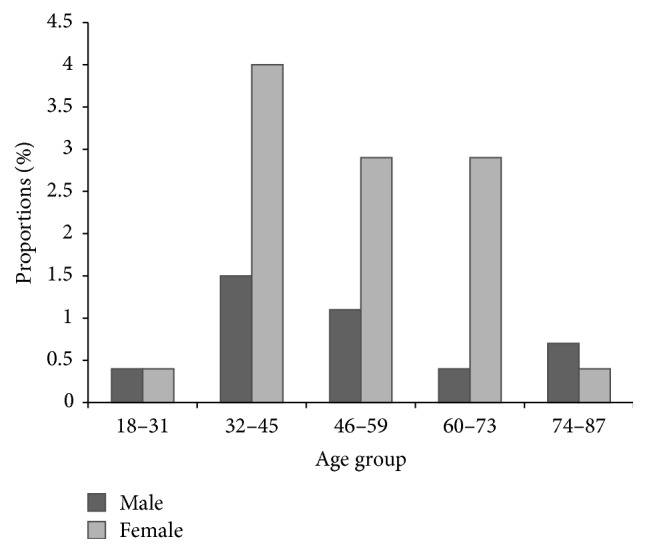
Age distribution of lymphoedema pathology according to stages in the examined individuals.

**Figure 3:**
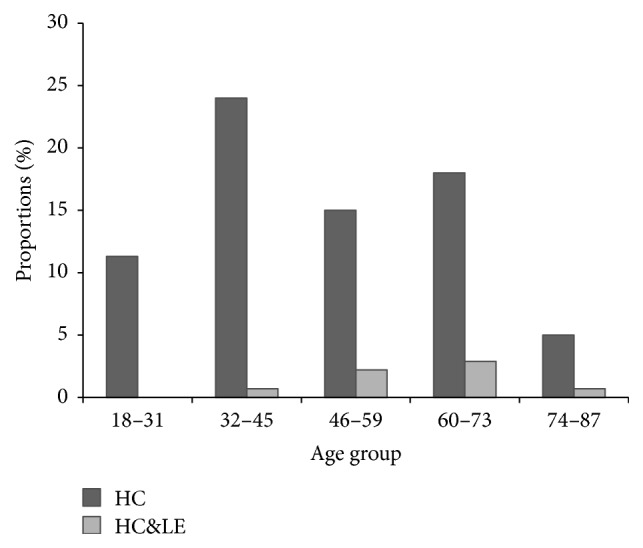
Age distribution of scrotal hydrocoele pathology according to stages in those examined individuals. HC: hydrocoele; HC&LE: hydrocoele and lymphoedema.

**Figure 4:**
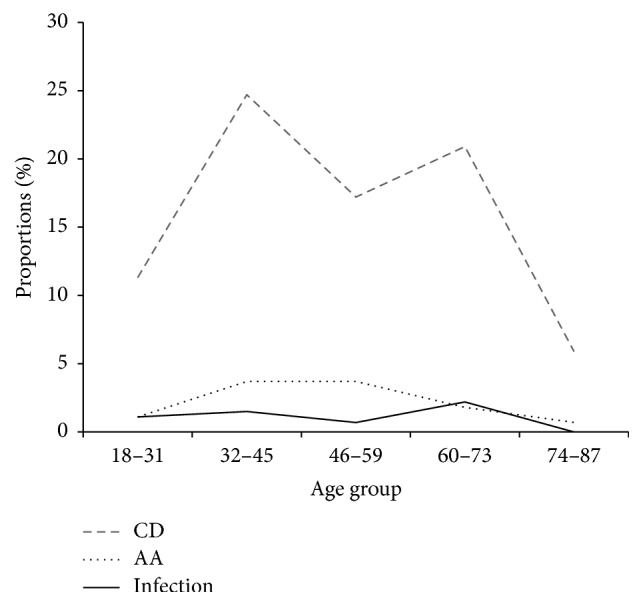
Acute and chronic clinical disease stratified into age groups. CD: chronic pathology (lymphedema, hydrocele, and lymphedema combined with hydrocele); AA: acute attacks (filarial fever); infection: circulating filarial antigen (CFA).

Females were observed to have a higher proportion of lymphoedema than males in the age group of 32–45 years (Figure 2). Figures 3 and 4 summarise the infection rates in the study group whereby 5.51% of the 272 individuals were CFA-positive and the majority of those were males (3.3%). Few of the confirmed CFA patients were females (2.21%). The proportions of scrotal hydrocele in males above 18 years old were high (73.2%) as compared to lymphoedema (15.8%). Conversely, the proportion of individuals with both lymphoedema and hydrocele was 5.51%.

## 4. Discussion

In our study, hydrocoele has been shown to be the main public health problem causing debilitation in males and similarly lymphoedema in females as observed elsewhere [12]. It has also been shown in our study that these conditions affect the 32–45-year age group. Recent studies focusing on the molecular mechanism regulating blood and lymphatic vessels growth have shown that vascular endothelial growth factors control angiogenesis and lymphangiogenesis in humans [13], which is a process of developing lymphangiectasia as a clinical disease. Similarly, expression of VEGF-A and VEGF-C has been shown to be upregulated by proinflammatory cytokines affecting the lymphatic vessels in males [13]. However, currently, there is limited data on the heterogeneity of the disease. Since the pathogenesis and development of lymphoedema remain largely unknown, there is a need for future studies to explore the role of genetics in relation to clinical phenotypes to better understand the disease aetiology and optimise the control strategies. The results of this study suggest that, during the era of lymphatic filariasis control of infections, the disease has shown a clear reduction of acute infection and morbidity rates compared to the baseline study, especially in the older age group. This could have been triggered by the host human-immune and parasite interaction leading to the clearance of acute infection [12]. On the other hand, the presence of adult worms and their secretions and the death of such adult worms may lead to dilatation of scrotal lymphatic vessel causing dysfunction and accumulation of protein-rich fluid in the tissue causing lymphangiectasia [13]. However, there is still ongoing LF transmission in some of the studied communities in the region and it is unlikely that the annual single dose can interrupt the transmission and therefore other strategies such as biannual administration of MDA can be applied to interrupt the transmission as observed in other studies. In Figure 4, young age groups (18–31 years) have increasing acute and chronic infections. A similar observation has been shown in Fiji [14]. These are the productive segment in the communities indicating a shifting trend of the clinical disease into the lower age groups because low-level transmission continues in those communities. This could be possibly due to some existing hotspots in the communities whereby mosquito vectors pick infection from humans and maintain the local transmission [15]. It is also likely that the postponement of the MDA distribution within the timely scheduled period could have halted reaching the desired coverage of at least 65% according to the WHO, hence leading to LF resurgence [14]. It is vital to expand the frequency of drug administration to a maximum of three doses per year, expand the age range of the target population, and improve health education to the community with the aim of increasing coverage within the targeted areas. Similarly, we should conduct an anthropological study to find out why transmission continues although MDA is in place, tracking factors associated with that, such as the incentive to drug distributors, health system roles in the district, timely availability of drugs, financial resources, and advocacy and training of distributors. The need for adequate financial and logistics resources is paramount to successfully achieve the targeted coverage and reach the end goal of the programme. Adequate resources and infrastructural support should be available to ensure timely availability and supply of the MDA drugs to reach the implementation units in endemic communities. Moreover, more evidence is needed to assess whether parasite genetic variability has implications in the effectiveness of diagnostic test, epidemiology, and control of the disease [16], as well as cross-reactivity of the ICT card to non-*W. bancrofti* filariae that could reinforce doubts on the validity of the current map of LF as observed in other studies [17]. Formulation of guidelines for LF coendemic areas assessment is therefore very important. It is worth also addressing issues related to the vector for successful control of LF. MDA in combination with other interventions such as insecticide-treated nets will improve reduction of infection and interrupt transmission of the infection in the communities. However, insecticide resistance could lead to a substantial increase and persistence of filarial infection incidence [18].

## 5. Conclusion

Our findings have shown a considerable reduction in filarial infection. However, there is clear evidence of ongoing transmission despite the 8 rounds of MDA using ivermectin and albendazole. It is unlikely that annual mass drug administration would interrupt filarial transmission; therefore, optimised strategies are needed to accelerate control and elimination of targets.

## Figures and Tables

**Table 1:** Sociodemographic characteristics of the study population.

Characteristics	Frequency (*N*)	Proportion (%)
*Age group in years *		
18–31	36	13.2
32–45	66	24.5
46–59	58	21.3
60–73	90	33.1
74–87	22	8.1
Sex ratio (female : male)	33 : 239	
*Occupation *		
Peasant farmers	102	37.5
Fishermen	170	62.5

*N*: number of individuals.
